# Complete plastid and mitochondrial genomes of the coralline marine red algae *Chiharaea americana* f. *americana* and *C. americana* f. *bodegensis* (Corallinaceae, Rhodophyta)

**DOI:** 10.1128/mra.00455-26

**Published:** 2026-06-04

**Authors:** Jose J. Aceves-Saldana, Aurpita Ahmed, Kimberly Alonzo, Moises Alvarez, Leilani M. Arroyo, Gisselle Avila, Lilly C. Baez, Kaeley N. Barbosa, Emily Bautista, Alfred Becerra, Aundrea Boykin, Aaron Braasch, Aaliyah Burton, Angelique A. Cabrera, Angelica Rosemarie F. Canque, Rafael Cantu, Diego Castaneda-Fuentes, William J. Clark, Jennifer De Jesus, Elizabeth De Lira, Ixchel C. Flores-Aranda, Moises Funtila, Paul W. Gabrielson, Julianna M. Gonzalez, Jadah S. Gustus, Hayli Hernandez-Celestino, Dain Hong, Jeffery R. Hughey, Aiden Ishimaru, Jamilah Ahmed Joseph, Hyemin Lee, Adrian I. Lopez, Mariah I. Marquez-Gonzalez, Angelita Martinez-Montesino, Carlosemilio Moreno, Ashley A. Ortiz, Leylani B. Ortiz, Emily A. Palomares, Fatima Pamatz-Angel, Max Payne, Marlen Pichardo, Naomi E. Pressey, Kennedy B. Rios Barragan, Genesis Rios Bustamante, Ulises Rios, Halie R. Roberts, Oscar L. Rodriguez, Destiny Sanchez, Lizbeth M. Sandoval, Jasmin Serrano, Aiden Valdez, Jaskaran S. Walia, Emma L. Winningham, on behalf of Hartnell College Genomics Group

**Affiliations:** 1 Division of Mathematics, Science, and Engineering, Hartnell College 17023, Salinas, California, USA; 2 Biology Department and Herbarium, University of North Carolina at Chapel Hill 2331, Chapel Hill, North Carolina, USA; 3 Department of Marine Biology, Pukyong National University 34998, Busan, South Korea; 4 Department of Marine Science, California State University, Seaside, California, USA; University of California Riverside, Riverside, California, USA

**Keywords:** bioinformatics, chloroplast genome, corallinales, Florideophyceae, high-throughput sequencing

## Abstract

We present the organellar genome sequences of *Chiharaea americana* f. *americana* and *C. americana* f. *bodegensis*. The plastid and mitochondrial genomes of f. *americana* and f. *bodegensis* are highly similar in length (plastid 180,947 vs 180,756, mitochondrial 25,923 vs 25,942), gene number (plastid 233 vs 234, mitochondrial 50), and organization.

## ANNOUNCEMENT

*Chiharaea* H.W. Johansen was erected to accommodate *Chiharaea bodegensis*, a marine coralline red alga from California exhibiting an extensive crustose base and erect fronds with 2–12 small irregularly branched intergenicula (1). Based on *rbc*L gene sequences, morphology, habitat, and biogeography, two more species were included in *Chiharaea*: *Chiharaea americana* (E.Y. Dawson and R.L. Steele) Martone, S.C. Lindstrom, K.A. Miller, and P.W. Gabrielson, and *Chiharaea silvae* (H.W. Johansen) Martone, S.C. Lindstrom, K.A. Miller, and P.W. Gabrielson (2). In a separate study based on analyses of multiple genes, *C. americana* and *C. bodegensis* were recognized as forms of the same species, as *Chiharaea americana* f. *americana* and *C. americana* f. *bodegensis* (3). To contribute to the genomic characterization of these two entities, their complete plastid and mitochondrial genomes were assembled and analyzed.

The specimens of f. *americana* and f. *bodegensis* were collected from Point George, Shaw Island, Washington, USA (48°19′28.2″N 123°03′54.1″W), voucher NCU 590280, and Hopkins Marine Station, Pacific Grove, California, USA (36°37′15.9″N 121°54′21.8″W), voucher NCU 593275, respectively. The DNA was extracted following published protocols (4, 5). The 150-bp paired-end libraries were constructed with the NEBNext Ultra II DNA Library Prep Kit (New England BioLabs) and sequenced on an Illumina NovaSeq 6000 (Illumina, Inc.) by Novogene Corporation Inc. The analyses generated 16,017,616 reads for f. *americana* and 12,312,206 for f. *bodegensis* that were filtered with the default BBDuk 1.0 (6) settings in Geneious Prime 2019.1.3 (Biomatters Limited), retaining 99.9% of the reads in both data sets. The organellar genomes were assembled *de novo* using filtered reads with default settings in MEGAHIT 1.2.9 (7). The genomes were extracted from the contig files and oriented to the beginning of the plastid genome of *Calliarthron tuberculosum* (GenBank accession number KC153978) and the mitochondrial genome of *Corallina chilensis* (GenBank accession number MK598844) using the alignment from a two-way Nucleotide BLAST+ 2.15.0 search using default settings (8). The genomes were circularized by removing the overlapping contig ends. The annotation was predicted in default mode with MFannot v3.0 (9) and refined with National Center for Biotechnology Information ORFfinder (https://www.ncbi.nlm.nih.gov/orffinder/) and Sequin 15.5 (10). Genic and intergenic mutations were determined with Geneious Prime. Nucleotide identities were calculated by BLAST+ searches using the default settings.

**Fig 1:**
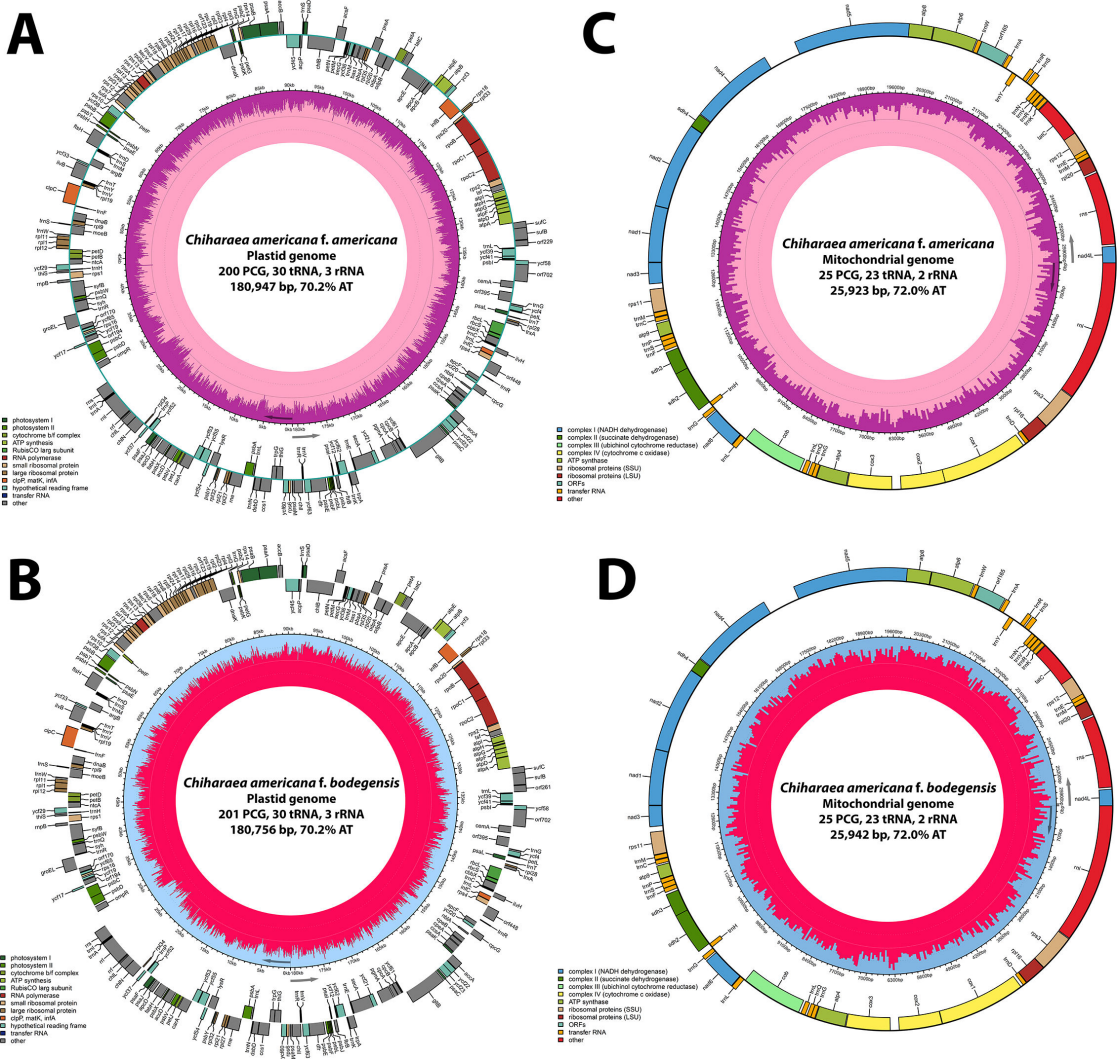
*Chiharaea americana* f. *americana* and *C. americana* f. *bodegensis* plastid (**A and B**) and mitochondrial genome maps (**C and D**). PCG = protein-coding genes. The genomes were mapped with Chloroplot 0.2.4 (11). The innermost ring displays the AT content and the direction of transcription, as indicated by the arrows. The final ring displays the genes. Genes transcribed clockwise are on the inside, while counterclockwise transcriptions are positioned on the outside. The color coding corresponds to genes of different groups, as listed in the key in the bottom left.

The complete circular plastid genomes of f. *americana* and f. *bodegensis* are 180,947 (212× coverage) and 180,756 bp (705× coverage) in length, respectively, with AT contents of 70.2%. The genomes contain 233 and 234 genes, including 200 and 201 protein-coding, respectively, 30 transfer RNA (tRNA), and 3 ribosomal RNA (rRNA) genes (Fig. 1A and B). The single gene difference is due to a hypothetical protein (orf456) present in f. *americana*, whereas in f. *bodegensis* the gene is split into two orfs (orf114 and orf282). The plastid genomes differ by 1,097 single-nucleotide polymorphisms (SNPs) (852 genic and 245 intergenic) and 16 indels (5 genic 1–21 bp in length, 11 intergenic 1–223 bp in length). The complete circular mitochondrial genomes of f. *americana* and f. *bodegensis* are 25,923 (163× coverage) and 25,942 bp (500× coverage) in length, respectively, with AT contents of 72.0%. The genomes contain 50 genes, including 25 protein-coding, 23 tRNA, and 2 rRNA genes (Fig. 1C and D). The mitochondrial genomes differ by 272 SNPs (227 genic and 45 intergenic) and 12 indels (5 genic 1–9 bp, 7 intergenic 1–7 bp). The f. *americana* and f. *bodegensis* plastid and mitochondrial genomes are 99.36% and 98.85% similar in identity, respectively.

## Data Availability

The complete chloroplast genome sequence of *Chiharaea americana* f. *americana* and *C. americana* f. *bodegensis* are available in GenBank under accession numbers PZ226319, PZ226320, PZ226321, and PZ226322. The associated BioProject, SRA, and BioSample numbers for f. *americana* are PRJNA1444732, SRS28569547, and SAMN56767199, respectively, and for f. *bodegensis* are PRJNA1444728, SRS28569450, and SAMN56766904, respectively.
